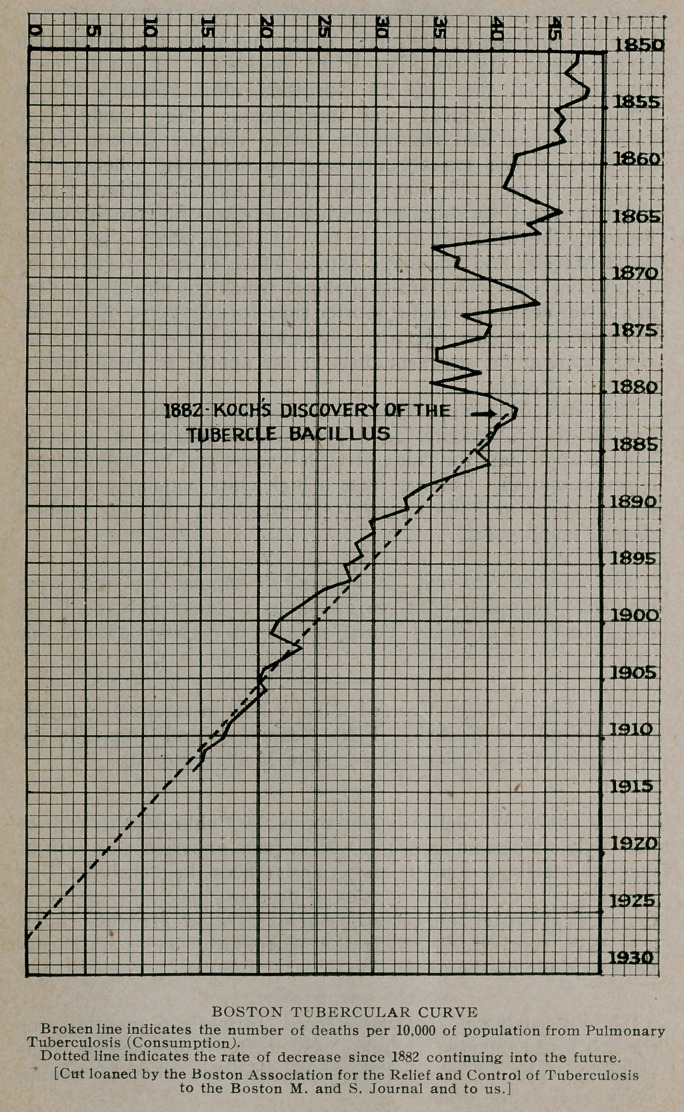# Topics of Public Interest

**Published:** 1914-03

**Authors:** 


### TOPICS OF PUBLIC INTEREST.

BOSTON TUBERCULAR CURVE

Broken line indicates the number of deaths per 10,000 of population from Pulmonary Tuberculosis (Consumption).

Dotted line indicates the rate of decrease since 1882 continuing into the future.

[Cut loaned by the Boston Association for the Relief and Control of Tuberculosis to the Boston M. and S. Journal and to us.]

Another Cancer Germ. A spirillum has been claimed by a Belgian as the cause of cancer. When taken from the intestine of the rat, fed to insects and these eaten by other rats, malignancy developes. This, in connection with various other observations, suggests that cancer in certain laboratory animals may be an entirely different disease than that of human beings.

Appointments. Carl Voegtlin has been appointed Professor of Pharmacology in the Hygienic Laboratory of the U. S. Public Health Service, to succeed Reid Hunt, now head of that department in Harvard.

---

30,000 Idiots and Feeble Minded in New York State, institutional accommodation for 4,000—Board of Charities report.

---

Cocaine Ruling. The Attorney General of New York State holds that a physician, unless he acts as a dispenser in the sense of a druggist, is not required to furnish a certificate giving name and address of patient, seller of drug, his own name and address and date and amount of drug. He must, however, keep a book record of all cocaines disposed of by him.

---

Virulent Vaccinia following vaccination soon after antityphoid vaccination, has been experienced by Prof. and Mrs. Wm. R. Shepard of Columbia.

---

The National Association For the Study and Prevention of Tuberculosis has adopted as its emblem a double red cross with all arms pointed.

---

Alvarenga Prize. The next award of about $180 will be made July 14, typewritten essays, accompanied by sealed envelope containing name and address, corresponding to moot on Mss., must be in hands of Dr. Thomas R. Neilson, Secretary of College of Physicians and Surgeons, 19 S. 22, Philadelphia, by May 1.

---

Bequest to Medical Society. The St. Louis Medical Society has received a legacy of nearly $50,000. We trust the precedent may be followed in our territory. Aside from the fact that medical societies are, generally speaking, worthy institutions, the endowment of the profession as a whole in any region would give it an influence over various ethical and economic problems that would be most wholesome.

---

Street Accidents. In New York City, automobiles killed 302 (149 children) in 1913; 221 (134 children) in 1912. Street cars killed 108 in 1913; 134 in 1912. Wagons killed 132 in 1913; 177 in 1912. In the State, outside New York City, the figures were, respectively, 150 and 127; 79 and 79; 32 and 28. The great increase in automobiles—there were 133,500 in the State in 1913—explains the growing list of automobile deaths and the diminution in the others. In Chicago, there were 3,589 fewer horses in 1913 than in 1912; 4,239 more automobiles and 612 more motor cycles. One moral is to keep children off the streets, another for adults to realize that the streets, between the curbs are no place for meditation. Probably a practical hint could be drawn if accurate statistics could be had of the number of deaths from automobiles driven by their owners and from those driven by chauffeurs, including taxicab drivers.

---

Mistake in Medicine. A nurse in the Utica Orphan Asylum gave medicine from the wrong bottle to six children on January 25, two dying as a result. Beside the obvious moral, it is rather too systematic to dose six patients from the same bottle, in one day.

---

Radium Appropriation. A bill has been introduced to buy $100,000 worth of radium for the New York State Institute for the Study of Malignant Disease. This is an excellent proposition. Radium is too expensive for most practitioners to carry in stock, and there is danger that, if purchased by private and semi-private institutions or combinations of physicians, it would be exploited for private ends. An adequate supply, bought by public money, and held in trust by a public institution, guarantees its use without this danger.

---

Lengthening of Course. Vanderbilt University, Tennessee, has extended its term to nine months, having been endowed from the Carnegie fund.

---

Fiske Prize Essay. The annual prize of $200 will be awarded to the best essayist on Causes, Symptoms and Early Recognition of Carcinoma Uteri. Essays must be submitted by May 1. For information, apply to Dr. Halsey DeWolf, 212 Benefed St., Providence (not Boston).

---

The Rochester General (Formerly City) Hospital celebrated its semi-centennial January 28.

---

X-Ray Treatment of Malignant Disease. This is not so recent as some writers have implied. Arthur F. Holding, *Am. Jour. of Roentgenology,* December, 1913, collated 3,134 cases thus treated from 1896 to 1909.

CANVASS OF "PRACTITIONERS." A committee of the Medical Society of the County of Erie, Dr. Grover W. Wende, Chairman, has issued a circular letter to its members requesting a report of all practitioners, by districts. The following method of classification is suggested: Care should, of course, be taken not to imply encroachment on medical practice by dentists, hospitals, nurses, druggists, barbers, etc., in good standing.

### GROUP I.

### PHYSICIANS AND SURGEONS HAVING THE DEGREE OF "M. D." AND LICENSED TO PRACTICE MEDICINE BY THE STATE OF NEW YORK.

Regular School.
Homeopathic School.
Eclectic School.

### GROUP II.

### INDIVIDUALS HOLDING A LICENSE OF SOME KIND FROM THE STATE OF NEW YORK.

Dentistry.
Osteopathy.
Pharmacy and Druggist.
Registered Nurse.
Barber.
Optometry (Optician).
Chiropody.

### INDIVIDUALS, CORPORATIONS, ETC., DOING BUSINESS WITH OFFICES.

Hospital.
Dispensary.
Institute (Sanitarium).
Patent Medicine.
Correspondence Concerns.
Manicure.
Beauty (Skin) Doctor.
Hair Dresser.
Deformity Appliances.
Chiropractic.
Cheiro-therapy.
Mechano-therapy.
Hydropathy (Baths).
Electric Baths (Magnetic Healer).
Massage (Rubbing).
Botanic Healer.
Cancer Cures.
Advertising Quack.
Bone Setter.

### RELIGIOUS METHODS.

Christian Science Healer.
Spiritualist.
Clairvoyant.
Fortune Teller.
Voodoo.
Witchcraft (Charms).
Emanualist.
New Thought.
Faith Healer.
Fetish Worship.
Oriental.
Metaphysician.

Col. Wm. C. Gorgas, M. D., has been appointed Surgeon-General U. S. A. This appointment should give universal satisfaction, as Col. Gorgas was in line for promotion by seniority and there can be no question as to his availability by those who claim that merit rather than seniority should be considered.

---

Suicide Prevention. The Chicago Salvation Army claims to have prevented 100 suicides in a year by its ministrations. Suicide, except in insanity and in instances in which the victim feels—rightly or wrongly need not be discussed here—that his removal from society is a public benefit, can almost always be prevented by human sympathy and help. Here we have an illustration of sentiment as a prophylactic factor, ranking in importance, so far as the death rate is concerned, with many highly scientific methods. Our own profession has almost the highest suicide rate of any occupation, except some that imply being "down and out."

---

Dental Dispensaries For Buffalo. The Board of Aldermen have passed a resolution establishing a central and two branch dispensaries. Through the generous offer of the Dental Department of the University of Buffalo the central dispensary will be equipped, housed and manned without cost to the city, so that it is estimated that this will cost only $1,000 a year, whereas the two branches, which are expected to do about half the total work, will cost about $2,500 apiece. This is a splendid charity, but it should be strictly limited to deserving cases and should not be allowed to pauperize those able to pay for services.

---

Exculpation of the Mayos. On page 356 et seq. of the January issue, we alluded to a criticism of the Mayos and stated "We are inclined to believe that the Mayos are in no way responsible for the postal card to which our contemporary takes exception" (a notice of trains leaving Chicago for Rochester, Minn., in time for clinics), "but that it is due to the enterprise of the passenger agent." We have received a file of copies of telegrams and correspondence fully supporting our judgment in the matter. This file also covers other matters of the same nature and shows that, so far from maintaining a publicity bureau, the Mayos have a definite system of preventing undue publicity This file may be consulted at our office by anyone interested.

---

Clinical Course at the Hotel Dieu, Paris. "Recent Notions on Maladies of the Pancreas and Spleen" will be presented by various lecturers at both morning and afternoon sessions,

April 6-18. The fee is 100 francs. Having taken a similar course on another subject, coprology, and having enjoyed, by courtesy, other opportunities for study at this institution, we strongly advise physicians expecting to take a European trip at this time, to include this course in their program. Application should be made to M. Deval, Chef de Laboratoire, Hotel Dieu, Paris.

---

AMERICAN SOCIETY FOR PHYSICIANS' STUDY TRAVELS. The first annual tour is scheduled to leave Atlantic City June 26, to visit various cities in the States and Canada and to end at Philadelphia, July 16. The party will be in Buffalo from the evening of June 29 to the morning of July 1, and will leave Niagara Falls early in the afternoon of July 2. First-class service is given throughout for a fee of $180. As travel is considerably more expensive here than in Europe, this is a very reasonable sum. Applications should be made to Dr. Albert Bernheim, 1225 Spruce street.

---

ACTING DENTAL SURGEONS. Twenty-eight vacancies now exist in the U. S. Army. Examinations will be held Monday, April 13, at several places. The pay is $150 per month, with perquisites. Appointments are for three years with promotion to the rank of Dental Surgeon, 1st Lt., at the end of that period, if qualified. Application should be made to the Surgeon-General at least two weeks before the examination.

---

EXAMINATION OF CANDIDATES FOR ASSISTANT SURGEON—United States Public Health Service. A board of commissioned medical officers will be convened to meet at the Bureau of Public Health Service, 3 B street, S. E., Washington, D. C., on Monday, March 9, 1914, at 10 o'clock A. M., for the purpose of examining candidates for admission to the grade of assistant surgeon in the Public Health Service. Examinations will be scheduled to be held at other stations of the Service at a later date.

After four years' service, assistant surgeons are entitled to examination for promotion to the grade of passed assistant surgeon.

Assistant surgeons receive $2,000, passed assistant surgeons $2,400, surgeons $3,000, senior surgeons $3,500, and assistant surgeon generals $4,000 a year. When quarters are not provided, commutation at the rate of $30, $40 and $50 a month, according to the grade, is allowed.

All grades receive longevity pay, 10 per cent. in addition to the regular salary for every five years' service up to 40 per cent. after twenty years' service.

The tenure of office is permanent. Officers traveling under orders are allowed actual expenses.

For invitation to appear before the board of examiners, address "Surgeon General, Public Health Service, Washington, D. C."

---

### The Bill to Restrict the Use of the Term Nurse.

The essential point of criticism in this bill is that by forbidding any but a registered, hospital graduate to call herself a nurse, it is virtually legislation against the dictionary. The word *nurse* as a general designation for anyone caring for a child or sick or injured person is practically as old as the English language, in fact some centuries older than English understandable by one who speaks that language today. An equivalent word occurs in most languages, ancient as well as modern, having the same general sense, so that it may fairly be stated that the word *nurse,* as a general designation, dates back to the dawn of civilization.

It is difficult to state just when the conception of the *trained nurse* originated. It is commonly dated back to the experience of the Crimean War, but, on the one hand, some degree of formal training of nurses, especially in sisterhoods, antedated this War by several centuries and, on the other hand, the *trained nurse* in the present sense, as a generally available aid to the physician and surgeon and as representative of a definite profession, did not arise till the seventies and eighties of the last century.

Sufficient time has elapsed, however, to produce a large, influential and capable body of active and retired trained nurses. The general requirements for the education, registration and legal control of this profession can now be best formulated by this profession for itself.

Objection to the bill in its present form is not an indication of prejudice for the untrained nurse, but fear that it will react to the detriment of the trained nurse. We doubt very much whether any court will rule that it is a legal offense to use a word in its accepted and long established sense. We feel very certain that no court will punish, to any sensible degree, anyone who calls herself a nurse, unless it can be clearly shown that she practiced deliberate deception by qualifying the word with such an adjective as "trained" or "hospital" or "registered." And we feel equally certain that any organized attempt to inforce the law will simply strengthen the opposition to the trained nurse. The contention that the laity do not understand the difference between a trained and an untrained nurse can scarcely be upheld.

Why not accept conditions as they exist, recognize that the trained nurse is alone susceptible of professional organization and development, and seek such legislative control of the noble profession of trained nurses, as their own experience dictates, letting the dictionary and the practical nurse alone?

### A New Abortion Bill

A bill has been introduced in the St. Louis Municipal Assembly. ' Any woman who shall, with intent to produce or promote her miscarriage or abortion, solicit any physician to administer to her any drug or substance whatsoever, or to use or employ any instrument or other means whatsoever, with intent thereby to procure her abortion or miscarriage, unless the same shall have been necessary to preserve her life, or shall have been advised by a physician to be necessary for that purpose, shall be guilty of a misdemeanor, and upon conviction shall be fined not less than twenty-five dollars nor more than two hundred and fifty dollars for each offense.

One of the most difficult problems in the control of abortion is that the principle of privileged communications shields the woman. She is nominally subject to severe punishment, so severe, indeed, that there is a natural hesitation toward inforcing it. Thus it usually happens that evidence of the crime is available only after she is dead. We believe that this legislation will accomplish practical results—not that it will entirely prevent abortion.

---

ADULTERATION AND MISBRANDING PUNISHED. The Dept of Agriculture reports recent convictions for selling dilute hydrochloric acid which did not come up to the standard; oil of bitter almonds deficient in hydrocyanic acid and containing chlorinated products; oil of cassia containing rosin and lead; oil of anise not conforming to the pharmacopœial standards.

---

TEMPORARY SANITARY SUPERVISORS have been appointed by Dr. Herman M. Biggs, State Commissioner of Health, at salaries of $4,000, as follows: Dr. Charles Duryee, Schenectady; Dr. Charles S. Prest, Waterford; Dr. John J. Mahoney, Jamestown; Dr. Frank S. Swan, Corning; Dr. T. Wood Clarke, Utica, and Dr. Otto R. Eichel, Buffalo.

---

AUTOMOBILE FRACTURES. The *Motor* states that over 2,200 fractures of the fore-arm occurred in 1913 from cranking. When one considers the vast amount of power generated by an automobile, the fact that a simple, inexpensive and reliable method of starting by pressure of a button, is a disgrace to our inventive genius. But, at least, one may avoid the inconvenience of exerting muscular power at a disadvantage and in the mud, and the danger of fracture, by a comparatively cheap and simple mechanic appliance.

---

UNION OF SOCIETIES. A movement is on foot to unite all of the general city and county societies of Rochester and Monroe Counties under one roof. This plan has been carried out in several cities to good advantage and involves economy, both of money, time and results.

---

PORNOGRAPHY IN BUFFALO. Recent accusations against Buffalo men and women whose lives were devoted to religious work, seem not even to have had the excuse of attempted blackmail nor, originally, to have indicated the anti-religious basis which they developed nor to have arisen as a part of that feud which narrow minds seem to consider inseparable from any religious, political or racial affiliation, unless in an individual sense. Even careful legal inquiry has, up to date, failed to show a spark of truth from which the shower of smut has started. But the episode has led to an exhibition of filthy art treasures—treasured because of their filthiness and included under the head of art with due apology—and, apparently, to the development of the keen commercial instinct which leads to the sale of memorial buttons when a President is killed as well as to the sale of all sorts of silly reminders of a great variety of events. We can make allowances for an occasional indulgence in onions, Limburger cheese and a passing, oral indecency, but we fail to comprehend the psychology of an endeavor to perpetuate such atmospheres. Some time ago a facetious friend laid on our desk an illustration of pornography which, it must be confessed, did have some humor beside the kind which is due to mere incongruity, and which, therefore, is supposed to attach to anything indecent. After his departure, we started to throw it into the waste basket, but recollected that that was not an ultimate disposition of satisfactory nature. An attempt to burn it revealed the fact that, with more care than is usually bestowed on art and literature of a higher kind, it was indelibly stamped on some silicious material, quite appropriate to its salacious nature As mental excrement, the water closet was an appropriate place, but we did not wish to clog the drain, especially with such incriminating testimony. The thing was hidden in a drawer and forgotten, except occasionally at night and in times of peril, when it was vividly remembered as something which surviving relatives would find as evidence of a side of our nature which we had carefully striven to conceal, even from ourself. It narrowly escaped production with other papers and articles of unobjectionable nature, before eyes that would have embarrassed us. Finally, after one of these narrow escapes, we devoted an hour's time with file and cutting forceps, to its destruction, and have breathed more freely since. Poronography is, most emphatically, not a survival from a state of savagery, nor can its rudiments be found in the lower animals, unless possibly in monkeys. It is a curious development of human psychology which seems to be synchronous with the development of æsthetics and morality—quite antagonistic characteristics. It does not even seem to be associated with sexual immorality in the ordinary physical form, unless quite accidentally, exceptions being as frequent as apparent indications of such association.

---

University Day. The University of Buffalo held its annual exercises at the Teck Theater, February 23. Dr. Charles William Dabney, president of the University of Cincinnati, delivered an address on the Municipal University.

---

Iola Sanatorium has issued its report for 1913, dedicated to the President of the Board of Managers, Dr. John F. W. Whitbeck. The Superintendent, Dr. Montgomery E. Leary, is to be congratulated on the excellent results obtained. This institution was established as a county hospital in 1910, and is located in Brighton, half a mile from the Rochester city line. The ground comprises twenty-five acres, consisting in part of a vegetable garden and hennery, which not only give wholesome employment to the patients but reduce the expense of maintenance. There are at present seven buildings, including power plant, etc. The supervisors have appropriated $75,000 to provide for enlargement of existing and erection of new buildings. X-Ray, pathologic and biologic laboratories will be included in the new equipment. 381 patients were treated in 1913, the accommodations being for 145 at a time. The capacity will be increased by 100, special attention being given to the needs of children. Only four times last year were there vacancies in the accommodations available. The per capita cost, per diem, was 2,095, the lowest of any comparable institution in the country.

---

Buffalo Electric Show will be held during the week of March 9, in Elmwood Music Hall. The central attraction will be an electric fountain 24 feet high and 20 feet in diameter—one of the largest ever erected—representing a volcanic crater, emitting a vapor which will be illuminated in various colors by searchlights beneath the floor, the top of the fountain being protected by a dome which will prevent the action of cross lights or the escape of rays from the searchlights. This arrangement represents an engineering triumph in the practical application of a study of angles of incidence, reflection and refraction. W. D'Arcy Ryan, Electric Engineer of the Panama-Pacific Exposition is the consulting engineer. The show is under the management of the Jovian League, and will be the most interesting and the largest show ever held outside of New York and Chicago. Every conceivable use of electricity will be exhibited, including its application to farming and housekeeping and certain developments of Wireless Conduction. The Thorarson Electric Manufacturing Co. of Chicago will show some developments of high tension wireless apparatus, as the cooking of food within a block of ice. The Robertson-Cataract Co. of Buffalo will have an interesting exhibit.

---

The Erie County Hospital graduated a class of fourteen nurses February 20.

---

New City Employees For Buffalo. The councilmen have amended the action of the aldermen by reducing the number of additional tuberculosis nurses to two instead of four. Otherwise they concur in the resolution for the appointment of two dental inspectors, two dentists, two assistants, two diagnosticians and eight clerks for the Health Department. A dancing hall inspector at $1,500 is also to be appointed. We trust that his duties will be held to include conferring some token of appreciation upon the two or three indecently dressed ladies who are usually in evidence at social functions. If they were always the same persons, the problem would be simpler.

## Figures and Tables

**Figure f1:**